# Postoperative analgesia and side effects of oral or injectable metamizole (dipyrone) in dogs and cats

**DOI:** 10.18849/ve.v8i4.671

**Published:** 2023-12-20

**Authors:** Jakub Stadnicki+, Mathieu Raillard+

**Keywords:** STRESS

## Abstract

**PICO question:**

In dogs and cats after surgery, does the peri-operative administration of injectable or oral metamizole (dipyrone) as opposed to no intervention result in lower postoperative pain scores or higher incidence of side effects?

**Clinical bottom line:**

**Category of research:**

Treatment.

**Number and type of study designs reviewed:**

Three prospective, randomised, blinded, clinical studies were critically reviewed.

**Strength of evidence:**

Weak.

**Outcomes reported:**

Variables assessed included: different pain assessment methods, metamizole dose required to reduce peri- and postoperative pain scores, changes in cardiovascular variables (heart rate, systolic, mean and diastolic blood pressure), changes in respiratory variables and variations in haematological and biochemical variables.

**Conclusion:**

In healthy cats and dogs undergoing ovariohysterectomy, the use of metamizole in the peri- and postoperative period was associated with some reduction in pain levels (i.e., lower pain score or reduced need for rescue analgesia). In dogs, metamizole alone provided insufficient analgesia. No study evaluating metamizole after orthopaedic surgery was found. No evidence suggested that using metamizole peri-operatively resulted in higher incidence of side effects.

## How to apply this evidence in practice

The application of evidence into practice should take into account multiple factors, not limited to: individual clinical expertise, patient’s circumstances and owners’ values, country, location or clinic where you work, the individual case in front of you, the availability of therapies and resources.

Knowledge Summaries are a resource to help reinforce or inform decision making. They do not override the responsibility or judgement of the practitioner to do what is best for the animal in their care.

## The evidence

Although metamizole (dipyrone) does not have a marketing authorisation for cats and dogs in the United Kingdom, its use is described in many other countries. There are tremendous regional differences in the attitude of physicians and veterinarians towards it, and a lot of confusion and myths, at least in Europe, in clinical practice.

Three randomised clinical trials were reviewed to identify if the administration of metamizole resulted in a reduced postoperative pain scores in healthy cats and dogs. Metamizole may contribute to reducing postoperative pain levels following ovariohysterectomy in those species. However, in dogs, metamizole is not appropriate as the sole analgesic in spay procedures. In cats and dogs, the dosages and intervals of administration of metamizole in the perioperative period require further elucidation. Side effects that were observed could not be attributed to metamizole.

## Summary of the evidence

**Imagawa et al. (2011):** 

Population:	Recruitment: Intact female dogs of different breeds and ages admitted to the surgery department for ovariohysterectomies. Criteria for eligibility and inclusion: Considered healthy by the investigators, without further detail. 10–35 kg. Criteria for exclusion and rejection: Known systemic or chronic disease. Other population information: Aged 1–6 years.
Sample size:	40 dogs.
Intervention details:	Dogs were assigned randomly to four intervention groups: NaCl (saline solution) 0.9% (placebo group) (n = 10). Metamizole 15 mg/kg (D15 group) (n = 10). Metamizole 25 mg/kg (D25 group) (n = 10). Metamizole 35 mg/kg (D35 group) (n = 10). Dosage and administration of interventions: Dogs received acepromazine, propofol, isoflurane and Ringer-lactated solution as part of their anaesthesia regimen. Metamizole or saline were all diluted to the same volume and administered intravenously (IV) before the end of the surgery and every 8 hours for 2 days postoperatively. Intramuscular (IM) tramadol 2 mg/kg used as a rescue analgesia.
Study design:	Prospective, randomised, blinded, clinical.
Outcome studied:	Level of analgesia after 0.9% saline solution and metamizole administration. Analgesia and sedation were evaluated and compared at regular intervals for 48 hours after the procedure using the following scoring systems: Visual analogue scale (VAS). A simple descriptive score. A scale based on behavioural patterns. Pulse rate, respiratory rate, systolic arterial blood pressure, diastolic arterial pressure, mean arterial pressure were also recorded at regular intervals for 48 hours after the procedure. Blood cortisol, catecholamines and selected haematological and biochemical variables were also measured in the post-operative phase. Rescue analgesia consumption was recorded.
Main Findings (relevant to PICO question)	Rescue analgesia was administered to all animals in the placebo group, to 7/10 animals in the D15 group and to 2/10 animals in both D25 and D35 group. Pain scores in dogs in the D25 and D35 groups were significantly lower when compared to the placebo group. There were no significant differences between the placebo and D15 groups. Vomiting was observed in 16/40 animals over the first 6 postoperative hours, four from the placebo group, five from the D15 group, four from the D25 group and three from the D35 group.
Limitations:	No sample size calculation or justification of the number of animals enrolled in the study; although authors report that a pilot study was performed on 25 animals, it seems to have been used to practice pain assessment rather than to justify the number of dogs to include in the analysis. Randomisation process not detailed. No detail on the blinding technique. Housing and husbandry of animals and animal care monitoring not thoroughly described.

**Zanuzzo et al. (2015):** 

Population:	Recruitment: Non-pregnant bitches scheduled for ovariohysterectomy. Criteria for eligibility and inclusion: Clinically healthy. Good temperament. Normal clinical pathology profile (complete blood cell count, serum biochemistry and venous blood gases). Criteria for exclusion and rejection: Abnormal laboratory values. Oestrus. Body Condition Score > 6/9. Other population information: Mean body weight (± SD): control group, 11.8 ± 4.2 kg and metamizole group,1 ± 3.6 kg. Mean age (± SD): control group, 30.4 ± 18.7 months and metamizole group, 11.1 ± 3.6 kg.
Sample size:	40 dogs.
Intervention details:	Dogs were assigned randomly to one of four intervention groups: Control group, CG (n = 10). Dipyrone (metamizole) group, DG (n = 10). Meloxicam group, MG (n = 10) (non pertinent to the current PICO question). Dipyrone-meloxicam group, DMG (n = 10) (non pertinent to the current PICO question). Dosage and administration of interventions: Dogs received pethidine, propofol, isoflurane and lactated Ringer’s solution as part of their anaesthesia regimen. Treatments were administered immediately following induction of anaesthesia as follows: CG: 0.1 mL/kg NaCl (saline solution) 0.9% intravenous (IV). DG: dipyrone 25 mg/kg IV. MG: meloxicam 0.2 mg/kg IV. DMG: meloxicam 0.2 mg/kg IV and metamizole 25 mg/kg IV in separate syringes.
Study design:	Prospective, randomised, blinded, clinical.
Outcome studied:	Intraoperative cardiovascular monitoring and anaesthetic requirements. Postoperative pain was evaluated using a modified Glasgow Composite Measure Pain Scale. Hyperalgesia assessed using mechanical nociceptive thresholds using an electronic Von Frey device in five different locations, using various flexible tips. Evaluations were performed before anaesthesia and at relevant time points post operatively for 24 hours after extubation. Rescue analgesia was administered if needed (cut-off criteria being described) and administration was recorded.
Main Findings (relevant to PICO question)	No difference in cardiovascular and anaesthetic requirements between groups. Significantly less animals needed rescue analgesia in the dipyrone group (DG) than in the control group. Following premedication with pethidine, metamizole (± meloxicam) reduced the need for postoperative treatment with morphine when compared with placebo or meloxicam alone.
Limitations:	No sample size calculation or justification of the number of animals enrolled in the study. Randomisation process not detailed. No detail on the blinding technique. Housing and husbandry of animals and animal care monitoring not thoroughly described.

**Teixeira et al. (2020):** 

Population:	Recruitment: Healthy, female cats presented for ovariohysterectomy. Criteria for eligibility and inclusion: Considered healthy after physical examination, haematological and biochemical analysis. Tolerance to manipulation evidenced by absence of fear-based aggression to blood collection, limb trichotomy and blood pressure measurement with Doppler sphygmomanometry. Other population information: Mean weight (± SD): Control 3.3 ± 0.5 kg, DIP1 2.9 ± 0.6 kg, DIP2 2.6 ± 0.4 kg, DIP3 2.8 ± 0.3 kg. Mean age (± SD): Control 26 ± 8.0 months, DIP1 24 ± 0.0 months, DIP2 22 ± 3.4 months, DIP3 24 ± 16.3 months.
Sample size:	28 cats.
Intervention details:	Cats were assigned randomly (drawing pieces of paper with group identification from a bag) to one of four intervention groups: Control group (n = 7). Metamizole at different time intervals (below) DIP1 (n = 7), DIP2 (n = 7) and DIP3 (n = 7). Four cats were removed (one from each group) for aggressive behaviour despite being docile at selection. Dosage and administration of interventions: (Cats received acepromazine, midazolam, pethidine, propofol, isoflurane as part of their anaesthesia regimen and tramadol at the end of the procedure and every 8 hours for 5 days postoperatively). Treatments were administered at the end of the procedure and for 5 days, as follows (metamizole was diluted in NaCl (saline solution) 0.9% to reach final volume of 1 mL): Control group: NaCl (saline solution) 0.9% 1 mL (intravenously) IV q8h. DIP1: Metamizole 25 mg/kg IV q24h. DIP 2: Metamizole 25 mg/kg IV q12h. DIP3: Metamizole 25 mg/kg IV q8h.
Study design:	Prospective, randomised, blinded, clinical.
Outcome studied:	Animals observed to detect adverse effects including vomiting, diarrhoea, salivation and anorexia. Effect of metamizole and tramadol used for 5 days on postoperative pain: Pain assessed using VAS, UNESP-Botucatu multidimensional pain scale and the Glasgow Feline Composite Measure Pain Scale by two evaluators blinded of the treatment preoperatively and at 3, 6, 12, 24, 36, 48 hours after extubation. Effect of metamizole and tramadol used for 5 days on haematological, biochemical and oxidative markers on erythrocytes: Venous blood was collected daily for 5 days and on day 10 to perform complete blood count and determine percentage of Heinz bodies. Serum was evaluated preoperatively on days 5 and 10. Superoxide dismutase, catalase, myeloperoxidase and lipoperoxidation were evaluated preoperatively and on days 3, 5 and 10.
Main Findings (relevant to PICO question)	No adverse effect observed. Administration of metamizole in cats, when used with tramadol, did not ensure better analgesia than tramadol alone (Teixeira et al.’s conclusion), yet rescue analgesia was used less often in groups receiving both metamizole and tramadol compared to groups receiving tramadol alone (control group). Metamizole did not cause significant biochemical alterations or oxidative damage to erythrocytes, although there were minor, ‘clinically irrelevant’, haematological differences between the groups.
Limitations:	Four cats (one per group) were excluded from the study based on fear-based aggression therefore making this study underpowered. Lack of group receiving only metamizole. No detail on the blinding technique. Housing and husbandry of animals and animal care monitoring not thoroughly described. Control cats had higher pain scores than DIP3 by UNESP-Botucatu and DIP2 and DIP3 by VAS 3 hours postoperatively; authors start the discussion stating that ‘metamizole slightly improved postoperative pain’ yet conclude that metamizole did not ensure better postoperative analgesia than tramadol alone.

## Appraisal, application and reflection

Metamizole (dipyrone) is an analgesic and antipyretic in human and veterinary medicine. Its pharmacokinetics and those of its two metabolites (4-aminoantipyrine and 4-methylaminoantipyrine) considered biologically significant are described in cats and dogs (Lebkowska-Wieruszewska et al., 2018; Giorgi et al., 2018; and Veras de Paula et al., 2023). Its main mechanism of action is not fully elucidated. Cyclooxygenase-2 inhibition (Campos et al., 1999), suppression of inflammation-induced nociception at a spinal cord level (Neugebauer et al., 1994), the involvement of endogenous opioids (Tortorici et al., 1996), have been described, among other modes of action (Silva et al., 2021). Its metabolites are reported to have anti-hyperalgesic effects through the activation of neuronal type 1 cannabinoid receptors (CB1) in peripheral tissue and by cyclic guanosine monophosphate (cGMP) activation and K_ATP_ (ATP-sensitive potassium channels) opening (Goncalves dos Santos et al., 2014).

The administration of metamizole can cause adverse events (Kötter et al., 2015). Whilst the drug is widely used in humans in many countries, it has been banned in others (e.g., The United Kingdom and The United States of America) because of safety concerns, in particular because of the risk of agranulocytosis. Genetics may play a role in incidence of side effects (Shah, 2019), which could explain the varying availability of the drug in different countries. This, in turn, possibly influences the restricted availability of metamizole on the veterinary market, explaining the limited number of publications and their geographical origin.

Multiple veterinary and human commercial preparations containing metamizole exist. Some contain metamizole alone, other contain metamizole and hyoscine hydrobromide. The various products use different excipients. In some countries, selected products containing metamizole and benzyl alcohol are registered and specifically contra-indicated in cats (e.g., Vetalgin® Vet., Sweden), presumably because of the excipient, whilst other products do not mention cats. The lack of evidence, coupled with the confusion caused by the numerous products and varying marketing authorisations, makes it challenging for clinicians to have a comprehensive understanding of metamizole use in dogs and cats.

We identified three articles partially addressing the following question: in dogs and cats after surgery, does the peri-operative administration of injectable or oral metamizole (dipyrone) as opposed to no intervention result in lower postoperative pain scores or higher incidence of side effects?

Animals enrolled in the three publications reported above were undergoing ovariohysterectomy. Although this procedure is a suitable model for visceral and acute, soft tissue, postoperative pain, conclusions on the relevance of metamizole at this stage cannot be extrapolated to other procedures. In particular, no conclusion can be made on the interest of metamizole following orthopaedic procedures. Lower postoperative pain scores were reported in one dog study at certain dosages (25 mg/kg and 35 mg/kg) (Imagawa et al., 2011) and in the cat study at certain time points with certain pain scoring scales only in animals receiving metamizole (Teixeira et al., 2020). However, it is important to point out that, in all studies, the administration of rescue analgesia was less in groups in which metamizole was administered. Although this study was not included in this Knowledge Summary because it focused on intraoperative nociception but did not evaluate the impact on post-operative analgesia, additional evidence suggests that the intraoperative use of metamizole in dogs could also offer some analgesia (Gorczak et al., 2022).

Side effects that were observed could not be attributed to metamizole (Imagawa et al., 2011; Zanuzzo et al. 2015; and Teixeira et al., 2020). Vomiting was observed in 16/40 animals over the first 6 postoperative hours in Imagawa et al. (2011), four from the placebo group, five from the metamizole 15 mg/kg group, four from the metamizole 25 mg/kg group and three from the metamizole 35 mg/kg group. This finding seems unrelated to metamizole since the incidence of vomiting in that study is rather high, but not very different among groups and from the placebo group. It could be due to the absence of a suitable, multimodal analgesic regiment in those animals for this procedure (spay).

Based on these studies, we conclude that the use of metamizole as the sole analgesic for ovariohysterectomy in dogs is inappropriate. Although metamizole alone was not administered to cats, we would exercise caution and extend this conclusion to cats as well. Comparisons among studies are challenging due to small sample size and variations in anaesthesia / analgesia protocols. Furthermore, all three studies have notable limitations. Metamizole may provide some degree of clinical analgesia following ovariohysterectomy in cats and dogs when administered in addition to other analgesics. Appropriate dosages and intervals of administration require elucidation.

## Methodology

**Search strategy:** 

Databases searched and dates covered:	CAB Abstracts via Web of Science (1910 – 2023 Week 40) PubMed (1966 – 2023 Week 40) Medline via Ovid (1946 – 2023 Week 40) Scopus (1970 – 2023 Week 40)
Search strategy:	CAB Abstracts: TOPIC: (Dog OR Dogs OR Canine OR cat OR cats OR feline) AND TOPIC: (metamizole OR dipyrone) AND TOPIC: (Pain OR Analg*) PubMed: ((Dog OR Dogs OR Canine OR Cat OR Cats OR Feline) AND (Metamizole OR Dipyrone) AND (Pain OR Analg*)) Medline: ((dog or dogs or canine or cat or cats or feline) and (metamizole or dipyrone) and (pain or analg*)).af. limit 1 to yr=”1946 - Current” (NB: “.af.” stands for All Fields) Scopus: ( TITLE-ABS-KEY ( dog OR dogs OR canine OR cat OR cats OR feline ) AND TITLE-ABS-KEY ( metamizole OR dipyrone ) AND TITLE-ABS-KEY ( pain OR analg* ) AND LANGUAGE ( "English" ) )
Dates searches performed:	04 Oct 2023

**Exclusion / inclusion criteria:** 

Exclusion:	Articles not available in English, single case reports, book chapters, conference proceedings, articles which did not answer the PICO question (e.g., metamizole vs other analgesics without a control group) and literature reviews.
Inclusion:	Available in English, not retracted.

**Search outcome:** 

Database	Number of results	Excluded –Not related to PICO	Excluded – Non-primary research	Excluded – Non-English publication	Excluded – Unable to access	Total relevant papers
CAB Abstracts	53	36	14	0	0	3
PubMed	50	47	0	0	0	3
Medline	30	19	5	3	0	3
Scopus	113	72	38	0	0	3
Total papers when duplicates removed						3

## ORCiD

Jakub Stadnicki: https://orcid.org/0009-0008-4164-8264 Mathieu Raillard: https://orcid.org/0000-0003-4057-9312

## Conflict of interest

The author declares no conflicts of interest.

## Disclaimer

Knowledge Summaries are a peer-reviewed article type which aims to answer a clinical question based on the best available current evidence. It does not override the responsibility of the practitioner. Informed decisions should be made by considering such factors as individual clinical expertise and judgement along with patient’s circumstances and owners’ values. Knowledge Summaries are a resource to help inform and any opinions expressed within the Knowledge Summaries are the author’s own and do not necessarily reflect the view of the RCVS Knowledge. Authors are responsible for the accuracy of the content. While the Editor and Publisher believe that all content herein are in accord with current recommendations and practice at the time of publication, they accept no legal responsibility for any errors or omissions, and make no warranty, express or implied, with respect to material contained within. For further information please refer to our Terms of Use.
